# Rules for the Prevention of Tuberculosis

**Published:** 1895-02

**Authors:** 


### Abstract.

### RULES FOR THE PREVENTION OF TUBERCULOSIS.

Comprehensive and efficient means should be adopted for the prevention of tuberculosis. I will recommend the following:

1. Educate the public to a proper understanding of the communicable character of tuberculosis. Teach the people how they can avoid contracting the disease themselves and how they can prevent transmitting it to others.

2. The promiscuous expectoration of consumptives should be prohibited. The sputum should be received into a 10 per cent. solution of carbolic acid, or an acid solution of bichloride of mercury, 1 to 1000. If at any time this be impracticable, the sputum may be collected on paper napkins or handkerchiefs, which must be burned before they become dry. Under no circumstances should a phthisical patient be allowed to spit on the floor or on the streets.

3. Let every physician employ systematic bacteriologic examination for the early diagnosis of the disease, and let us inaugurate compulsory registration of all cases of tuberculosis.

4. It should be made compulsory to have careful and thorough disinfection of all houses, apartments, penal and reformatory institutions, carriages, street and railway cars, steamships, theaters, churches, and the like, which have been exposed to infection from phthisical patients.

For disinfection of rooms I would recommend sulphur acid gas, obtained by burning one ounce of sulphur to every ten cubic feet of space, chlorin gas to saturation. Shut all the doors, windows and crevices for four hours, then let in the fresh air and scrub the walls, floor and articles of furniture with the acid bichloride solution (bicloride of mercury, ʒ ij ; tartaric acid, ℥ij to the gallon of water, or ʒ ij each of $HgCl_2$ and permanganate of potassium to the gallon. Remove all wall paper if it cannot be washed or painted. Thoroughly boil or steam all bedding, carpets, curtains, and the like, for at least one hour.

5. Under no circumstances should the stools of tuberculous patients be emptied into sewers until they have been thoroughly disinfected. The intestinal glands are frequently implicated in tuberculosis, and the dejecta often teem with bacilli ; therefore, all discharges should be received into a solution of eight ounces of carbolic acid to the gallon, or four ounces of chloride of lime to the gallon of water.

6. Enact regulations prohibiting tuberculous individuals from following vocations that may expose others to the danger of infection. The sputum may dry on their beards or clothing and then be disseminated. For the same reason, consumptives should avoid kissing, or even hand-shaking, to protect those near and dear to them. All dishes and drinking cups should be used by the patient exclusively, and should never be mingled with those in use by other members of the family. The promiscuous use of public drinking cups in schools, cars, streets and churches cannot be too severely condemned, as contagion is possible from this practice.

7. Tuberculous mothers should not nurse their children. In fact, consumptive people should not be permitted to marry.

8. There should be established careful scientific examination, under city and state control, of all milk, meat and other articles of food sold. All animals suffering from tuberculosis, anthrax, septicemia, glanders, cattle plague, sheep pox, swine plague, foot and mouth disease, acute pneumonia, actinomycosis, dropsy and rabies should be killed and at once *cremated.*

9. Consumptives should always be isolated and there should be established, under state control, public hospitals and sanitaria for the segregation and isolation of the consumptive poor, where they could live under the best hygienic laws, receive proper food and judicious medicament.

10. All persons having died of tuberculosis should be at once wrapped in sheets wrung out of bichloride solution, and cremated as soon as practicable. If this be not possible, then they should be buried with quicklime, as the bacilli do not die with their host, but have been found in cemeteries from two to twenty-five years after inhumation.

Is it not our duty to prevent the ravages of tuberculosis and thereby save over 150,000 lives annually in the United States? Are we not bound by our obligations to ameliorate suffering and prevent loss of life, and this can be accomplished by isolation, proper hygiene and disinfection. That tuberculosis is contagious —even from man to wife—no one but the ancient, non-progressive physician will deny.—*Dr. Winslow Anderson in the Journal of the American Medical Association.*

